# Assessment of 11 β-Hydroxysteroid Dehydrogenase Type I Activity in Patients With Psoriasis Vulgaris: A Novel Insight Into the Pathogenesis of the Disease

**DOI:** 10.7759/cureus.66262

**Published:** 2024-08-06

**Authors:** Ahmed Nassar, Nermeen Abd El Fattah, Ahmed Afify, Nashwa El-Khazragy, Mahira El Sayed

**Affiliations:** 1 Department of Dermatology, Andrology and Venereology, Ain Shams University, Cairo, EGY; 2 Department of Clinical Pathology-Hematology, Ain Shams Medical Research Institute (MASRI) Ain Shams University, Cairo, EGY

**Keywords:** stress, case-control study, steroidogenesis, inflammation, psoriasis

## Abstract

Background

Psoriasis is a relapsing dermatologic disease with a complex multifactorial etiology. Accumulating evidence has established the presence of cutaneous steroidogenesis with 11 β-hydroxysteroid dehydrogenase (11βHSD) enzyme being the most important final step of this pathway. This enzyme can control local levels of activated glucocorticoids (GCs) in the skin, which is the key to maintaining healthy skin.

Methods

This case-control study was conducted to evaluate 11βHSD1 level in psoriasis patients, in both lesional and non-lesional skin, compare it to controls, and correlate its activity with the Psoriasis Area and Severity Index (PASI) and the Perceived Stress Scale (PSS).

Results

A significant decrease of 11βHSD1 level in psoriasis patients compared to healthy controls was observed. In addition, decreased 11βHSD1 level was observed in lesional compared to non-lesional skin in psoriasis patients. There was no significant correlation between the enzyme levels and PASI score or PSS score in patients with psoriasis. However, the PSS score was negatively correlated with 11βHSD1 level in healthy controls. Further histopathological assessment revealed that lower enzyme levels were associated with greater epidermal acanthosis and inflammation.

Conclusion

This shows the role of 11βHSD1 in controlling psoriatic inflammation, including the degree of epidermal proliferation, which might reveal the complex symphony of psoriasis pathogenesis.

## Introduction

Psoriasis is a chronic relapsing dermatologic disease resulting from the interaction between genetic and environmental factors with a complex multifactorial etiology [1]. Skin is not the only organ which is clinically affected by psoriasis. There is robust evidence that supports the involvement of other systems with increased risk of comorbidities such as psoriatic arthritis, metabolic syndrome, and depression [2].

Being one of the common skin disorders associated with psychological distress, psoriasis proves to have a complicated relationship with psychological distress and mental disorders. Thus, disturbance of the hypothalamic-pituitary-adrenal (HPA) axis activity, which is one of the physiological factors associated with stress, was suspected to be one of the causative links between psoriasis and stress [3].

Many studies showed that HPA axis over-activity can influence, not only the mood but also the skin. Corticotropin-releasing hormone (CRH) can stimulate local cutaneous cytokine production like IL-6 and IL-11, and increase the expression of adhesion molecules on keratinocytes like intercellular adhesion molecule-1 (ICAM-1) [4-6]. Additionally, CRH activates the pro-inflammatory nuclear factor kappa-B (NF-𝜅B) pathway, which is a major part of the inflammatory response in psoriasis [7].

In the case of psoriasis, a statistically significant increase of CRH receptor type 1 (CRH-R1) was found in psoriatic lesions, and a positive correlation between the severity of PASI and CRH-R1 expression was identified as well [8]. Moreover, a defect in the HPA axis was detected in psoriasis. Studies showed that, in the case of psoriasis, the HPA axis usually responds to stressors with deficient cortisol levels, which might predispose to exacerbation of the disease [9,10].

In 1996, Slominski et al. [11] presented the idea of cutaneous steroidogenesis. Later, the skin was found to have its own HPA axis analog which can produce cortisol locally [12], which makes the skin capable of responding to biological, physical, and/or psychological stressors.

Hannen et al. [13] provided additional proof that the skin can produce glucocorticoids (GC) locally by epidermal keratinocytes which protects the skin against external insults. In addition, they detected defective synthesis of GC and decreased expression of nuclear GC receptors (GR) in lesional and perilesional psoriatic skin. Thus, they suggested that defective downstream immunosuppressive signaling, including CRH and GC, underlies the development of inflammatory skin conditions including psoriasis [12-14]. Conversely, overstimulation of the local GC pathway results in skin infection, weakened epidermal barrier, and atrophy of the skin [15].

11 β-hydroxysteroid dehydrogenase (11βHSD) is one of the important enzymes that can modulate GC availability and activity in the skin at a pre-receptor level independently of systemic levels. It acts by interconverting active cortisol and inactive cortisone by its two isozymes. 11βHSD1 (which activates cortisol), but not 11βHSD2 (which inactivates cortisol), was found to be expressed in human skin in both epidermal keratinocytes and dermal fibroblasts [16-18].

Accordingly, the balance between 11βHSD1 and 11βHSD2 isoenzymes is crucial for maintaining healthy skin since increased or insufficient levels of GCs or their receptor responses can impair skin barrier function [19,20]. The expression of 11βHSD1 in psoriasis was investigated before, being a representative of epidermal hyperproliferation in inflammatory skin conditions. In that study, 11βHSD1 expression in the epidermis was decreased in the lesional skin of psoriasis vulgaris [21]. However, this study focused on the epidermis and keratinocyte proliferation only.

Herein, we investigated 11βHSD1 level as part of the dysfunction in psoriasis patients, in both lesional and non-lesional skin, and correlated its activity with the Psoriasis Area and Severity Index (PASI) and the perceived stress scale (PSS). This can explain part of the defect of steroidogenesis in the skin of psoriasis patients, provide a different insight into the interplay between various immune pathways involved in the pathogenesis of psoriasis, and might show a novel link between psoriasis and psychological stress.

## Materials and methods

This is a case-control study that was conducted from March 2022 to February 2023. Thirty-one adult patients with a dermatologist-confirmed diagnosis of psoriasis vulgaris and 30 age-matched healthy controls, with a negative family history of psoriasis, were included in the study. Psoriasis vulgaris patients were divided into three equal groups based on the degree of severity of psoriasis as measured by PASI score. Patients were excluded if they had any inflammatory skin disease other than psoriasis, pustular or erythrodermic psoriasis, psoriatic arthritis, patients using topical or systemic treatment, or patients with concurrent psychiatric illness or on psychotropic medication.

Each subject underwent: full history taking including demographic and clinical characteristics, assessment of the severity of the disease by PASI, assessment of psychological stress by the 10-item version of the PSS-10, and assessment of the impact of psoriasis on life quality by dermatology life quality index (DLQI). Two 3 mm punch skin biopsies were collected from both lesional and non-lesional skin of patients, and only one 3 mm punch skin biopsy from controls. Skin biopsy from the non-lesional skin and control subjects were taken from a non-sun-exposed site (upper inner arm or upper thigh) to exclude any changes in the enzyme level due to exposure to ultraviolet rays. Tissues were rinsed in ice-cold phosphate buffer saline (PBS) PH 7.4 and stored at ≤-20°C until analyzed. Afterwards, Tissue Ruptor II (Qiagen, Hilden, Germany) was used for disruption and homogenization of tissues. The mixture is then centrifugated for 20 minutes at 4,000 rpm. Finally, the cell supernatant is collected for RNA extraction. The 11βHSD1 was measured in tissue homogenate using the enzyme-linked immunosorbent assay (ELISA). The assay was performed by 11βHSD1 ELISA kit, cat no: SG-15236 (SinoGene Biotechnology, Hangzhou, China).

Consequently, five more patients were recruited, and two punch skin biopsies were collected from each patient from a psoriatic lesion on a non-sun-exposed area. One biopsy was collected on PBS for assessment of the enzyme level, while the other was fixated in formalin for histopathological examination (H&E).

Data management and analysis

Data analysis was performed using the Statistical Package for Social Sciences, version 25.0 (Released 2017. IBM Corp., Armonk, NY). Numerical variables were compared using the student t-test, Mann-Whitney U-test, or Kruskal Wallis test depending on the distribution of raw data. The chi-square (χ2) test or Fisher’s exact test was used to compare categorical variables. Correlation between numerical variables was assessed by Pearson or Spearman correlation coefficient according to data distribution. The receiver operating characteristic (ROC) curve was used to evaluate the sensitivity and specificity for quantitative diagnostic measures that categorize cases into one of two groups. A significance level of p < 0.05 was used in all tests.

## Results

The mean age was 42.9 ± 14.52 years among the patients' group and 39.4 ± 14.41 years among the controls group. Demographic and clinical characteristics among the study patients are shown in Tables 1, 2, respectively. No significant differences were found between the two groups regarding age, body mass index (BMI), PSS score, and severity (p>0.05).

**Table 1:** Demographic and clinical characteristics among the study patients N: Number, PASI: Psoriasis area and severity index, DLQI: Dermatology life and quality index, PSS: Perceived stress scale

		N	Percent
Gender	Male	25	80.6
	Female	6	19.4
Marital status	Single	5	16.1
	Married	26	83.9
Smoking	No	11	35.5
	Yes	20	64.5
Family history	Positive	1	3.2
	Negative	30	96.8
Past history	Positive	7	22.6
	Negative	24	77.4
Drug history	Positive	5	16.1
	Negative	26	83.9
Fitzpatrick skin phototype	II	1	3.2
	III	11	35.5
	IV	18	58.1
	V	1	3.2
PASI severity	Mild	10	32.3
	Moderate	10	32.3
	Severe	11	35.5
DLQI severity	Small	8	25.8
	Moderate	5	16.1
	Very large	7	22.6
	Extremely large	11	35.5
PSS severity	Low	8	25.9
	Moderate	14	45.2
	High	9	29.0
Location of first lesion	Scalp	11	35.5
	Upper limb	10	32.3
	Trunk	5	16.1
	Lower limb	5	16.1
Pruiritis	No	7	22.6
	Yes	24	77.4
Previous treatments	Topical	21	67.7
	Multiple	10	32.3
Efficiency of previous treatment	No	15	48.4
	Yes	16	51.6
Longest period of remission (months)	<1	23	74.2
	1-3m	4	12.9
	>3m	4	12.9

**Table 2:** Clinical characteristics among study patients IQR: Interquartile range, SD: Standard deviation, BMI: Body mass index, PASI: Psoriasis area and severity index, DLQI: Dermatology life and quality index, PSS: Perceived stress scale

	Mean	±SD	Minimum	Maximum	Median	IQR	
Age	42.90	14.52	20.00	68.00			
BMI	27.84	5.10	21.00	43.00			
Age of first lesion	36.16	15.81	17.00	71.00	30.0	21.0	49.0
Body surface area	24.61	21.24	4.00	75.00	12.0	8.0	37.0
PASI	12.03	10.89	2.30	46.00	9.0	4.6	14.9
DLQI	15.10	9.38	2.00	30.00	15.0	5.0	24.0
PSS	20.65	9.64	3.00	38.00	21.0	12.0	29.0

Patients recalled several triggering factors for psoriasis, the most common was the change of season; 22 patients (71%) reported flare of their lesions in winter. Additional provoking factors are shown in Table 3.

**Table 3:** Triggering factors for psoriasis

	N	Percent
Stress	10	32.3
	21	67.7
Mechanical trauma (Koebner phenomenon)	17	54.8
	14	45.2
Change of seasons	9	29.0
	22	71.0
Infections	24	77.4
	7	22.6
Increase body weight	29	93.5
	2	6.5
Sun exposure	20	64.5
	11	35.5

Assessment of the enzyme level revealed that lesional enzyme level among psoriasis patients ranged from 7.6 to 1,030.6 ng/g tissue, with a mean value of 270.18 ± 203.61 ng/g tissue, and the non-lesional enzyme level ranged from 184.6 to 1,304.6 ng/g tissue, with a mean value 503.76 ± 231.44 ng/g tissue. On the other hand, the mean enzyme level among the study controls was 831.13± 183.89 ng/g tissue and ranged from 414.6 to 1,088.6 ng/g tissue.

It was clear that the lesional enzyme level was notably lower in psoriasis cases compared to healthy controls (p=0.001). Moreover, the non-lesional enzyme level showed a significant decrease in psoriasis cases compared to controls (p=0.001) (Table 4). Additionally, among psoriasis patients, there was a significant decrease in the enzyme level in psoriatic lesions compared to non-lesional sites (p=0.001) (Table 5).

**Table 4:** Comparison between cases and controls as regard enzyme level, PSS score, and baseline characteristics *Chi-square test, **Fisher exact test, ‡Student t-test, ‡‡Mann-Whitney U test IQR: Interquartile range, HS: Highly significant, NS: Non-significant, BMI: Body mass index, PSS: Perceived stress scale

	Group										P-value	Sig
	Case					Control						
	Mean	±SD	Median	IQR^^^		Mean	±SD	Median	IQR			
Age	42.90	14.52	46.0	31.0	53.0	39.40	14.41	41.0	26.0	51.0	0.348^‡^	NS
BMI	27.84	5.10	26.2	24.0	30.0	25.59	4.44	24.2	22.5	28.7	0.072^‡^	NS
PSS	20.65	9.64	21.0	12.0	29.0	20.60	8.52	24.0	15.0	27.0	0.96^‡^	NS
Lesional enzyme	270.18	203.61	281.6	106.6	386.6	831.13	183.89	834.6	706.6	988.6	0.001^‡‡^	HS
Non-lesional enzyme	503.76	231.44	504.6	324.6	577.6	831.13	183.89	834.6	706.6	988.6	0.001^‡‡^	HS

**Table 5:** Comparison between lesional and non-lesional enzyme among study patients *Wilcoxon Signed Ranks Test IQR: Interquartile range, SD: Standard deviation, HS: Highly significant

	Mean	±SD	Median	IQR		P-value	Sig
Lesional enzyme	270.18	203.61	281.6	106.6	386.6	0.001*	HS
Non-lesional enzyme	503.76	231.44	504.6	324.6	577.6		

Neither the lesional nor the non-lesional enzyme levels in psoriasis patients were correlated with PASI, DLQI, or PSS scores (p>0.05). Conversely, the enzyme level among controls was negatively correlated with PSS scores (r=-0.795, p<0.001).

**Figure 1:**
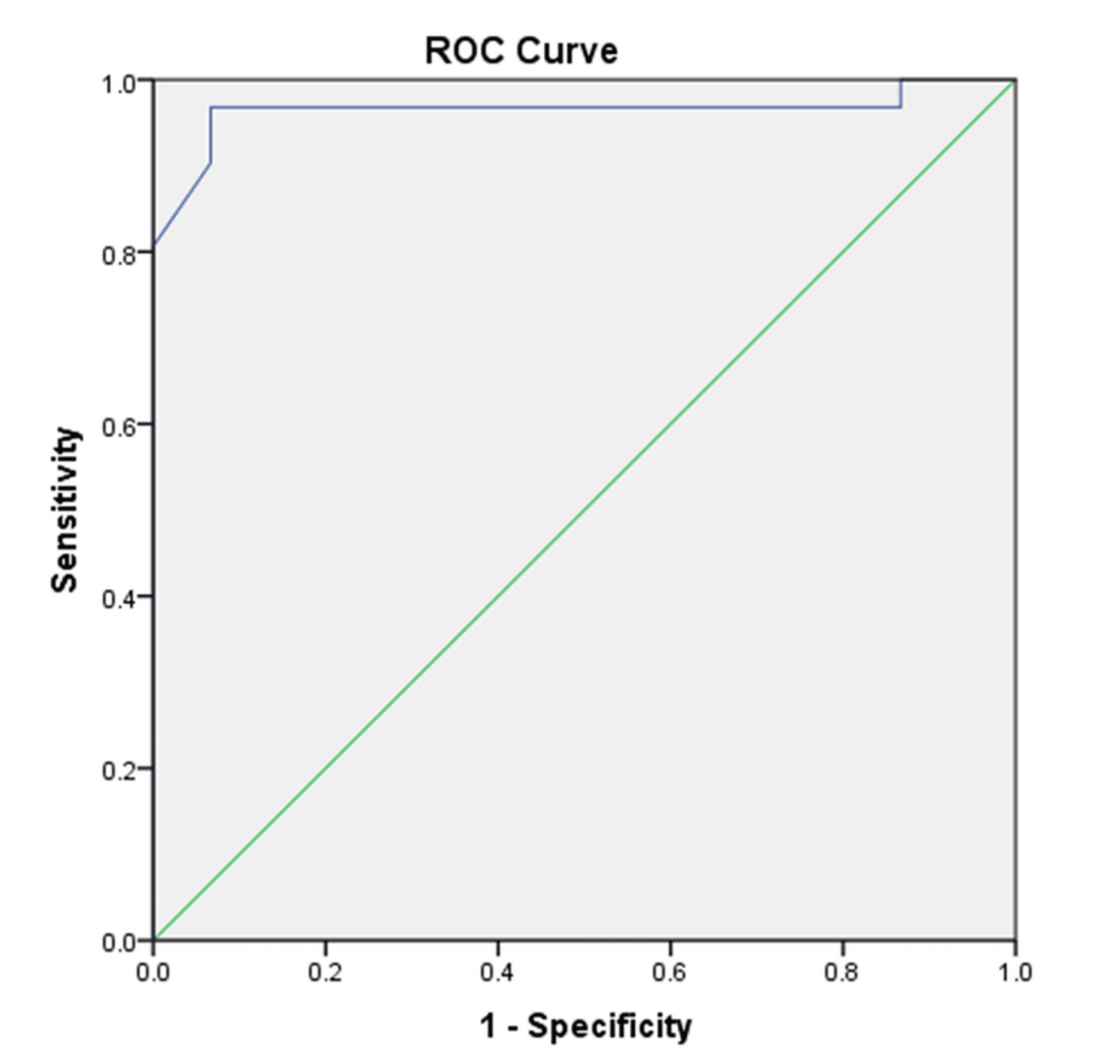
ROC curve using non-lesional enzyme to differentiate between cases and controls ROC: receiver operating characteristic

It came to our attention that there was a significant positive correlation between the non-lesional enzyme level and the longest period of remission as recalled by the patients (r=0.362, p=0.045). Thus, ROC analysis was performed to determine the value of the non-lesional enzyme level to detect susceptibility to develop psoriasis, or supposedly any inflammatory skin condition, at a cutoff level ≤600.1, with 80.6%, 93.3%, and 88.2% sensitivity, specificity, and accuracy, respectively (Figure 1).

**Figure 2:**
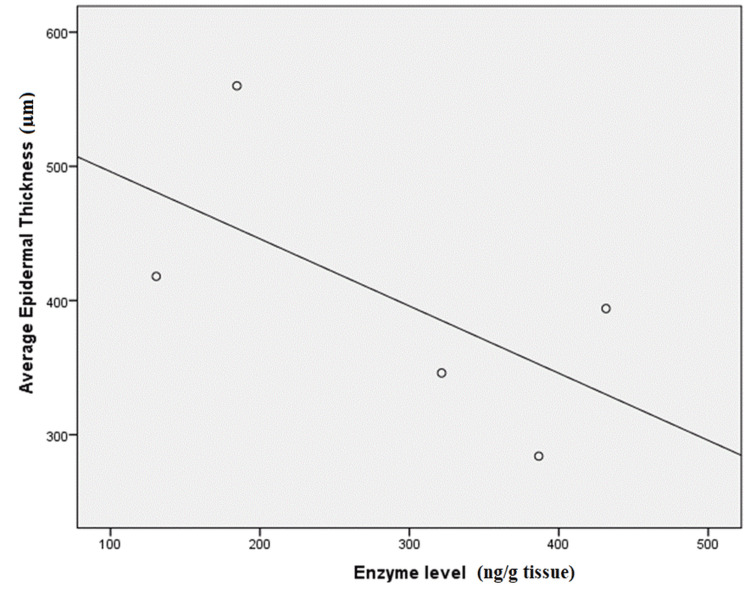
Correlations between lesional enzyme level and average epidermal thickness among cases

On further assessment of the histopathological H&E sections, there was a strong negative correlation between the enzyme level and the average epidermal thickness (r= -0.6) (Figure 2); however, this was not statistically significant due to the small sample size. Of note, the epidermal thickness was measured from the bottom of the rete ridge to the bottom of the stratum corneum. Average epidermal thickness was calculated as the mean of three different measurements.

**Figure 3:**
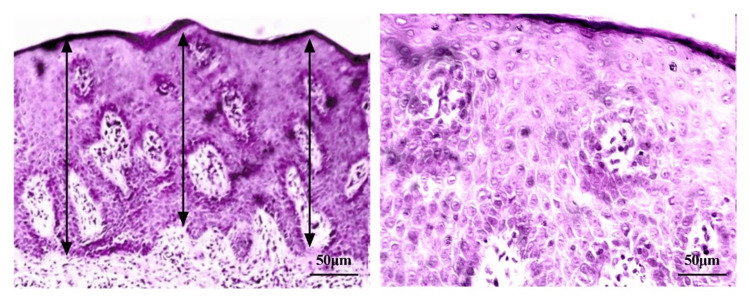
A lesional skin of a psoriasis patient showing a significant increase in epidermal thickness (acanthosis) and inflammatory cell density (stained with H&E) The image magnifications are x10 (left side) and x40 (right side). The black arrows represent the measurement of average epidermal thickness.

**Figure 4:**
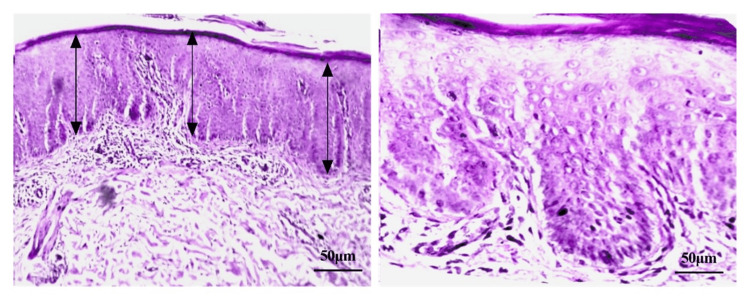
A lesional skin of a psoriasis patient showing less increase in epidermal thickness (acanthosis) and inflammatory cell density (stained with H&E) The image magnifications are x10 (left side) and x40 (right side). The black arrows represent the measurement of average epidermal thickness.

Discrepancies in the average epidermal thickness when compared to the lesional enzyme level are shown in Figures 3, 4. A significant increase in the average epidermal thickness (acanthosis) (560 µm) and inflammatory cell density can be seen in Figure 3 with very low enzyme level (184.6 ng/g tissue) from the same lesion of one patient, compared to a less increase in the average epidermal thickness (284 µm) and inflammatory cell density in Figure 4 with relatively higher enzyme level (386.6 ng/g tissue) from the same lesion of another patient.

## Discussion

The pathogenesis of psoriasis depends on an interplay between different genetic and environmental factors that are responsible for the disruption of skin homeostasis. Studies proved that several cytokines are responsible for the maintenance of the inflammatory cascade. Yet, the initial stimulus for inducing inflammation is unknown [1,22]. Daily, skin is subjected to several local stressors which demand a special mechanism to maintain homeostasis. Local cutaneous steroidogenesis is one of the main suggested mechanisms [23].

Our study showed that the 11βHSD1 enzyme level, being a vital part of this homeostatic mechanism, is dysfunctional in the case of psoriasis. The enzyme level, whether in lesional or non-lesional skin, was significantly lower in psoriasis cases compared to controls. Furthermore, the 11βHSD1 enzyme in the lesional skin showed significantly lower levels in comparison to the non-lesional skin. No significant differences were found between the two study groups regarding age, BMI, and PSS severity (p>0.05), proving that both groups were comparable in all other aspects that might affect the enzyme level.

In agreement with the current study, Baser et al. [24] revealed that the 11βHSD1 enzyme was significantly reduced in the lesional skin of 20 patients compared to 15 controls [24]. Likewise, Tognetti et al. [25] examined skin biopsies from 10 patients with psoriasis vulgaris. 11βHSD1 was markedly downregulated in lesional psoriatic skin, suggesting the presence of dysfunction in local steroidogenesis in these patients [25].

In 2017, Boudon et al. [26] showed that in vitro inhibition of 11βHSD1 was shown to increase both keratinocyte and fibroblast proliferation. This suggests that the defect in local steroidogenesis in the lesional skin of psoriasis patients might contribute to the development of keratinocyte hyper-proliferation [18,26].

Trying to test the diagnostic accuracy of the 11βHSD1 enzyme to differentiate predisposed individuals from healthy controls, ROC curve analysis was performed for the non-lesional enzyme level compared to controls. It revealed that at a cutoff level ≤600.1 ng/g tissue, with high sensitivity and specificity, the 11βHSD1 enzyme in uninvolved healthy skin can be used as a marker to identify susceptibility for psoriasis. However, given that the enzyme level could be affected by multiple factors, it would be difficult to assess the enzyme level solely in healthy individuals as a marker for diagnosis of psoriasis. Yet, this cutoff value might reflect the susceptibility to psoriasis in people with a positive family history of the disease, or the liability of certain body parts in patients to develop psoriasis later in the course of the disease. On a similar note, there was a significant positive correlation between the non-lesional enzyme level and the longest period of remission (r=0.362, p=0.045). This supports our theory about the major role of 11βHSD1 enzyme activity in controlling local inflammation in psoriasis patients.

Surprisingly, no correlation was detected between the lesional or the non-lesional enzyme level in patients and the PASI score (p>0.05). Although the 11βHSD1 obviously affects the severity of inflammation in a single psoriatic lesion, the calculation of the PASI score depends on other factors, like the surface area involved, which might alter the total score. Furthermore, the enzyme level in a specific skin area could be altered by several factors including ultraviolet irradiation, enhancing 11βHSD1 expression in sun-exposed areas [25].

To our knowledge, this is the first study to assess the correlation between the 11βHSD1 enzyme and PSS score in healthy controls. A systematic review by Gregory et al. [27] focused on the use of 11βHSD1 inhibitors in diseases known to be associated with abnormalities of HPA axis function [27]. Our findings suggest a relation between cutaneous 11βHSD1 enzyme level and the degree of stress as perceived by the person. This sheds light on the defect in the stress coping mechanisms, including HPA axis dysfunction, and its possible role in the pathogenesis of psoriasis vulgaris [28]. Despite the absence of a significant correlation between the enzyme level and PSS score in patients, the suggested defect could act only as a trigger for initiating the inflammatory cascade after which many elements may influence the enzyme level, including keratinocyte proliferation and fibroblast function.

Further assessment by histopathological examination displayed a strong negative correlation between the enzyme level and the average epidermal thickness in the same psoriatic lesion (r= -0.6). In line with the current study, Terao and Katayama [18] revealed that the 11βHSD1 staining score is significantly lower in psoriasis vulgaris and correlates negatively with epidermal thickness [18]. Similarly, Sarkar et al. [29] showed that the increase in 11βHSD1 mRNA expression can be correlated with the progression of epidermal differentiation (p < 0.05) [29]. So far, it is still debatable whether the change of 11βHSD1 expression is the cause or the result of keratinocyte hyperproliferation and decreased differentiation associated with psoriasis vulgaris [18,30].

## Conclusions

Our study showed that the 11βHSD1 enzyme level, being a vital part of the skin homeostatic mechanism, is dysfunctional in the case of psoriasis. This can explain the role of 11βHSD1 in controlling psoriatic inflammation which might reveal a new part of the complex symphony of psoriasis pathogenesis. Accordingly, modifying 11βHSD1 enzyme activity might be a new potential therapeutic target to control the degree of epidermal proliferation and the severity of inflammatory infiltrate in the case of psoriasis.

The current study was limited by a small sample size, being a single-center study. Further studies with larger sample sizes are needed to confirm our results and to identify a clearer image of the enzyme's role in the pathogenesis of disease.
